# Utilizing combinatorial engineering to develop Tie2 targeting antagonistic angiopoetin-2 ligands as candidates for anti-angiogenesis therapy

**DOI:** 10.18632/oncotarget.16827

**Published:** 2017-04-04

**Authors:** Tomer Shlamkovich, Lidan Aharon, William A. Barton, Niv Papo

**Affiliations:** ^1^ Department of Biotechnology Engineering, and the National Institute of Biotechnology in the Negev, Ben-Gurion University of the Negev, Beer-Sheva, Israel; ^2^ Virginia Commonwealth University, Department of Biochemistry and Molecular Biology, Richmond, Virginia, United States of America

**Keywords:** protein-protein interactions, protein engineering, directed evolution, angiogenesis, antagonistic activity

## Abstract

In many human cancers, the receptor tyrosine kinase (RTK) Tie2 plays important roles in mediating proliferation, survival, migration and angiogenesis. Thus, molecules that could potently inhibit activation of the Tie2 receptor would have a significant impact on cancer therapy. Nevertheless, attempts to develop Tie2-targeted inhibitors have met with little success, and there is currently no FDA-approved therapeutic selectively targeting Tie2. We used a combinatorial protein engineering approach to develop a new generation of angiopoietin (Ang)2-derived Tie2 antagonists as potential cancer therapeutics and as tools to study angiogenesis. The construct for designing a yeast surface display (YSD) library of potential antagonists was an Ang2 binding domain (Ang2-BD) that retains Tie2 binding ability but prevents ligand multimerization and receptor dimerization and activation. This mutant library was then screened by quantitative high-throughput flow cytometric sorting to identify Ang2-BD variants with increased expression, stability and affinity to Tie2. The selected variants were recombinantly expressed and showed high affinity to soluble and cellular Tie2 and strongly inhibited both Tie2 phosphorylation and endothelial capillary tube formation and cell invasion compared to the parental Ang2-BD. The significance of the study lies in the insight it provides into the sequence-structure-function relationships and mechanism of action of the antagonistic Ang mutants. The approach of using a natural protein ligand as a molecular scaffold for engineering high-affinity agents can be applied to other ligands to create functional protein antagonists against additional biomedical targets.

## INTRODUCTION

The receptor tyrosine kinases (RTKs) Tie2 and Tie1 and their angiopoietin (Ang) endothelial growth factor ligands (in the human, Ang1–Ang4) are known to be involved in the formation of blood vessels—both developmental and pathological [1–6]. In studies to delineate the exact roles of these kinases and their ligands, the greater part of the research effort to date has been directed to Tie2 and Ang1 and Ang2 [7, 8]. It is now known that Ang1 mediates endothelial cell growth, proliferation, and neovascularization through binding and activation of Tie2 [9]. The role of Ang2 appears to be somewhat more complex: in some cases it serves as a “decoy” to prevent Ang1-Tie2 binding, whereas in others it forms a complex with Tie2 to facilitate angiogenesis [4, 7, 8].

Ang1 and Ang2 are multimerized by their coiled-coil and N-terminal domains, with their receptor binding activity residing in their carboxy-terminal fibrinogen-like domains [10–13]. Upon binding to Ang1 multimers, the Tie2 receptor oligomerizes, bringing its kinase domains into close proximity and thereby enabling the kinase domains to phosphorylate each other to promote cell proliferation, migration, and sprouting [14]. Activation of Tie2 via tyrosine phosphorylation initiates the phosphatidylinositol 3-kinase (PI3K)-Akt pathway, which leads to Akt and MAPK/ERK signaling and hence to both endothelial cell survival and migration phenotypes [15, 16].

Accumulating evidence has revealed a significant correlation between the expression of Ang1 and Ang2 in tumor invasion and metastasis in a variety of human cancers [17–19]. Similarly, Tie2 overexpression has been implicated in breast, ovarian, and liver cancers and in glioblastomas [20], with Tie2 reaching its highest levels in the peripheral neovascular endothelium of invasive tumors [21]. In light of the above, it seems likely that molecules targeting Tie2 would show promise as cancer therapeutics and diagnostics and also as tools to study Tie2 activation and inhibition during angiogenesis. Nonetheless, the work that has been done on molecules targeting the Tie2 receptor has met with very little success: there are currently no FDA-approved therapeutics that selectively target Tie2, and there are only a few Tie2-kinase domain inhibitors in pre-clinical trials, probably due to the toxicity or off-target effects of these inhibitors [22]. In a different approach, angiopoietin antagonists, including the soluble recombinant extracellular domains of the Tie2 receptor, have been investigated as potential anti-tumor therapeutic agents [23–25]. For example, blocking angiopoietin function by binding the ligand to a soluble Tie2 ectodomain (Tie2-Fc) inhibited tumorigenesis and neovascularization in postnatal rats, presumably due to depletion of the available ligand. However, given that multiple angiopoietin isoforms (i.e., Ang1–4) can bind to Tie2, it is not clear whether this effect is mediated by the inhibition of multiple isoforms or of a single isoform, and the former scenario may lead to unwanted effects. Yet another approach is based on therapeutic monoclonal antibodies and antibody fragments that target growth factors and their receptors. These strategies have indeed met with clinical success, but they are not without their limitations. To date, there is only one peptide-Fc fusion protein (peptibody) that neutralizes the interaction between Ang1 and Ang2 with Tie2; this peptibody has exhibited potency in systemic xenograft models and in a rat corneal model of angiogenesis [26, 27]. Similarly, there are only a few human monoclonal antibodies targeting the Ang2 ligand in early-stage clinical trials [28–30].

In light of the research described above, it seems that the approaches that hold the most promise for developing therapeutics are those aimed at inhibiting ligand-mediated receptor activation by receptor antagonists derived from natural ligands [31–42]. Our strategy is therefore to use natural ligands as the basis for engineering protein-protein interactions, since natural effectors will interact with ligand-binding residues on the receptors, without the limitations associated with antibodies and peptibodies, which would not necessarily bind these functionally important epitopes. An additional advantage of ligand-based antagonists is that they are inherently likely to be more specific than kinase domain small molecule inhibitors; this advantage is conferred by their large interaction surface, which involves both the highly conserved active site and different surrounding target-interacting residues. However, the less than optimal ligand binding affinity and specificity, expression yield, and stability have impeded the development of ligand-based antagonists as cancer therapeutics: to date, only a few such therapeutics have advanced to clinical trials [43, 44].

Previous work has, however, provided an indication as to how the potential of ligand-based antagonists could be realized: In particular, a rational engineering approach was applied to transform the natural multimeric Ang1 agonist into a monomeric high-affinity Tie2 antagonist [9]. The ligand multimerization was abolished by mutating two amino acids on residues critical for Ang1 multimerization in the superclustering domain (SCD) of the molecules (i.e., C41S and C54S). The Ang1_C41S,C54S_ variant bound Tie2 well but did not multimerize properly and, consequently, could not activate Tie2 [9]. Since the diminished affinity of Ang1_C41S,C54S_ (relative to wild-type Ang1) for Tie2 presumably resulted in a low inhibitory effect of the mutant in Ang1-mediated processes [3,45], we reasoned that the development of efficacious monomeric Ang-based antagonists would be contingent on improving their affinity for Tie2.

Taking advantage of the high sequence and structural similarities between Ang1 and the natural Tie2 antagonist, Ang2, we began by constructing a monomeric version of Ang2, namely, the Ang2-binding domain (Ang2-BD). The next step in our strategy was to engineer the natural monomeric Ang2-BD for higher affinity binding and subsequently improved antagonism to Tie2. To this end, by using yeast surface display (YSD) to randomly screen Ang2 variants [46], with increasingly stringent sorts against Tie2, we identified mutants with the highest stabilities and target affinities. The final steps were to demonstrate that these high-affinity Tie2 antagonists are potent inhibitors of Tie2 signaling *in vitro* and are able to inhibit angiogenesis in cell-based models.

## RESULTS

### Affinity maturation of Ang2-BD YSD libraries

**Figure 1:**
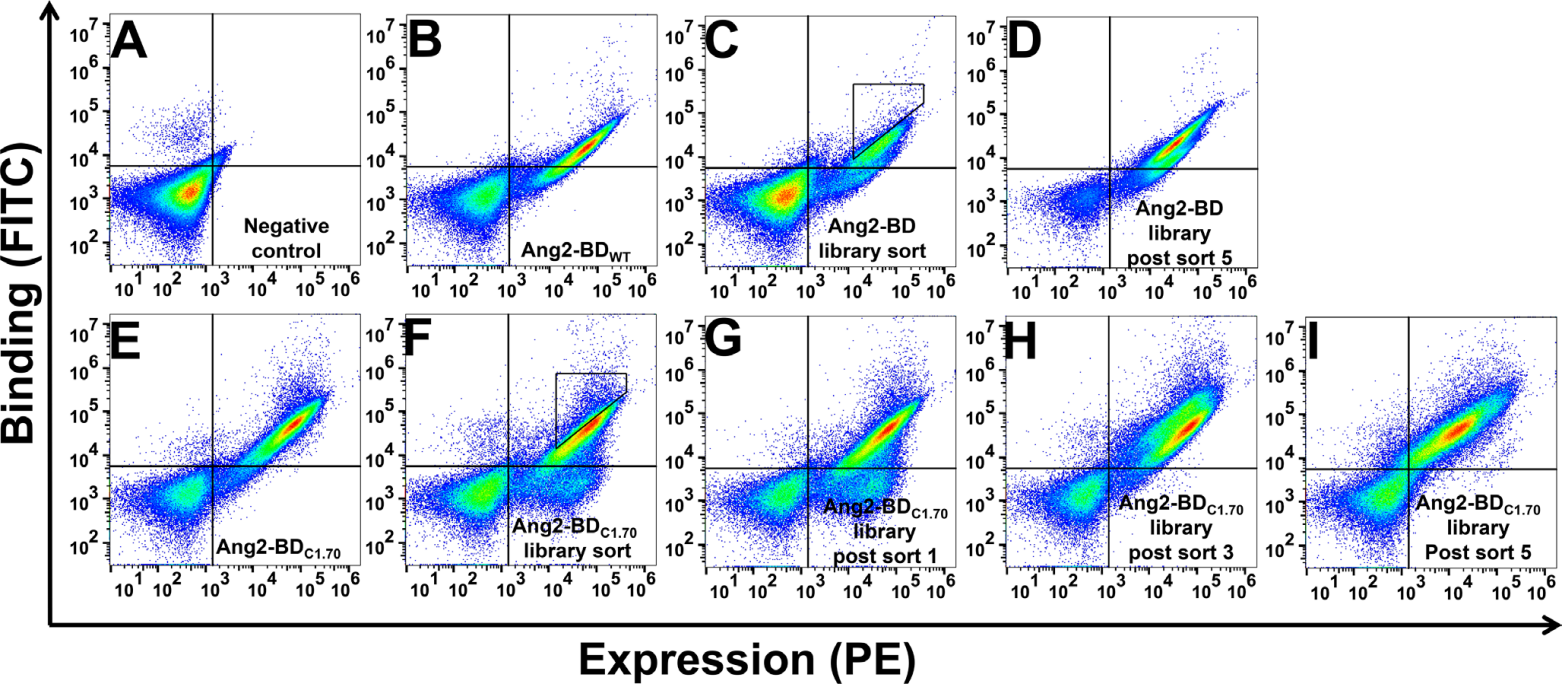
Screening of first- and second-generation Ang2-BD libraries against soluble Tie2 Shown is a FACS analysis of yeast expressing Ang2-BD. (**A**) Negative control. (**B**) Ang2-BD_WT_ expression and binding of Tie2 (10 nM). (**C**) Ang2-BD library expression and binding of Tie2 (10 nM). (**D**) Ang2-BD library expression and binding of Tie2 (10 nM) after five rounds of sorting. (**E**) Ang2-BD_C1.70_ expression and binding of Tie2 (5 nM). (**F**) Ang2-BD_C1.70_ library sort expression and binding of Tie2 (5 nM). (**G**–**I**) Ang2-BD_C1.70_ library expression and binding of Tie2 (5 nM) after sorts 1, 3 and 5, respectively. Sorts 2–5 were conducted using gates similar to the one shown in panel F.

Wild-type Ang2-BD (Ang2-BD_WT_) was created as the starting point for affinity maturation towards recombinant human (rh)Tie2. It was first necessary to test the compatibility of Ang2-BD with the YSD system that was to be used subsequently as a platform for the creation of the Ang2-BD library and affinity maturation towards Tie2. To this end, Ang2-BD was cloned into a YSD plasmid (pCTCON) and presented on the yeast cell surface as a fusion to agglutinin proteins. High yeast display and Tie2 binding levels were detected for Ang2-BD by staining with fluorescently labeled antibodies as compared to unstained controls. A 12-amino-acid linker (LPDKPLAFQDPS) was added between the cMyc tag and Ang2-BD to prevent steric hindrance between the two antibodies (Supplementary Figure 1). A yeast-displayed library in which random mutations were introduced to the *Ang2-BD* gene was generated using error-prone PCR, with 2–9 mutations per clone and a yield of approximately 6 × 10^6^ transformants. This Ang2-BD first-generation library, enriched for expression, was subjected to four additional rounds of sorting with decreasing concentrations of Tie2 (Figure 1A–1D). The sorting gates are shown in Figure 1C for the selection of clones with high affinity relative to their expression. The expression and binding of the YSD library at the beginning and the end of the sorting process are shown in Figure 1C and 1D, respectively.

### Isolation of clones from the first-generation library with improved binding affinity towards Tie2

To identify specific Ang2-BD variants with improved Tie2 binding affinity, 70 individual clones were isolated from the fifth sort of the affinity maturation. Most of the clones showed a 50% increase in affinity relative to Ang2-BD_WT_, with clone C1.70 showing the highest (2.5-fold) increase in affinity (Supplementary Figure 2). Not surprisingly, sequencing analysis of individual clones isolated from this first-generation library revealed mutations, such as K432N, I434T, N467K, F469L, N470D and Y475H, that are located within the Ang2-BD/Tie2 binding interface. In particular, in clone 70 (C1.70), which had the highest affinity towards Tie2, there were three mutations in its binding interface and one additional mutation in close proximity to the Ang2-BD-Tie2 interface (Supplementary Table 1).

### Isolation from the second-generation library of clones showing further improvement in binding affinity towards Tie2

**Figure 2:**
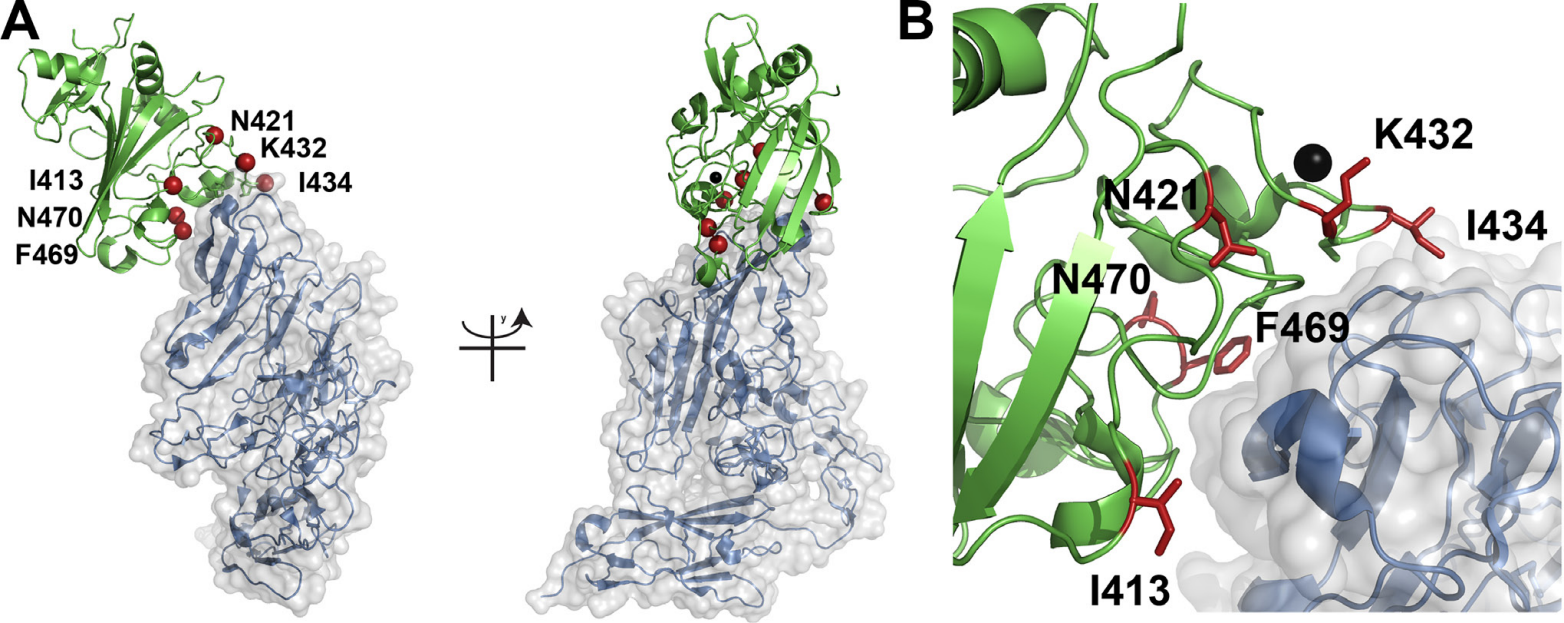
Localization of Ang2-BD mutations (**A**) The Ang2/Tie2 complex (PDB 2GY7) is shown in cartoon format with Ang2 in green and Tie2 in blue. The location of mutations in Ang2-BD_C2.36_ are shown as red spheres at the Cα atom and labelled accordingly. The panel on the left is rotated 90 degrees along the y-axis relative to the image on the right. (**B**) A close-up view of the Ang2/Tie2 binding interface highlighting the location of the six mutations. A calcium atom is shown in space filling format while the mutations are in ball-and-stick.

Screening a second-generation library based on the Ang2-BD_C1.70_ variant (Figure 1E) resulted in a diversity of approximately 8 × 10^6^ transformants. Five rounds of sorting, starting with library expression enrichment and followed by target screening at decreasing concentrations of Tie2, were performed (Figure 1F–1I). Following the sorting, a significant shift of the library towards high affinity binding was clearly evident (Figure 1I). With the aim to isolate variants with improved binding affinities from the second-generation library, 60 individual clones were isolated, sequenced and tested for their binding affinity towards Tie2 (Supplementary Table 2). Based on the sequencing results and the increase in Tie2 binding affinity, two clones, Ang2-BD_C1.70_ and Ang2-BD_C2.36_, were chosen for their increased binding affinity (2.5- and 17-fold as compared to Ang2-BD_WT_, respectively) towards Tie2 (Supplementary Figure 3). Figure 2 shows the positions that were mutated in wild-type Ang2-BD to generate Ang2-BD_C2.36_. Ang2-BD_C1.70_ and Ang2-BD_C2.36_ were purified and used in the subsequent experiments.

### Production and biochemical evaluation of soluble Ang2-BD variants

**Figure 3:**
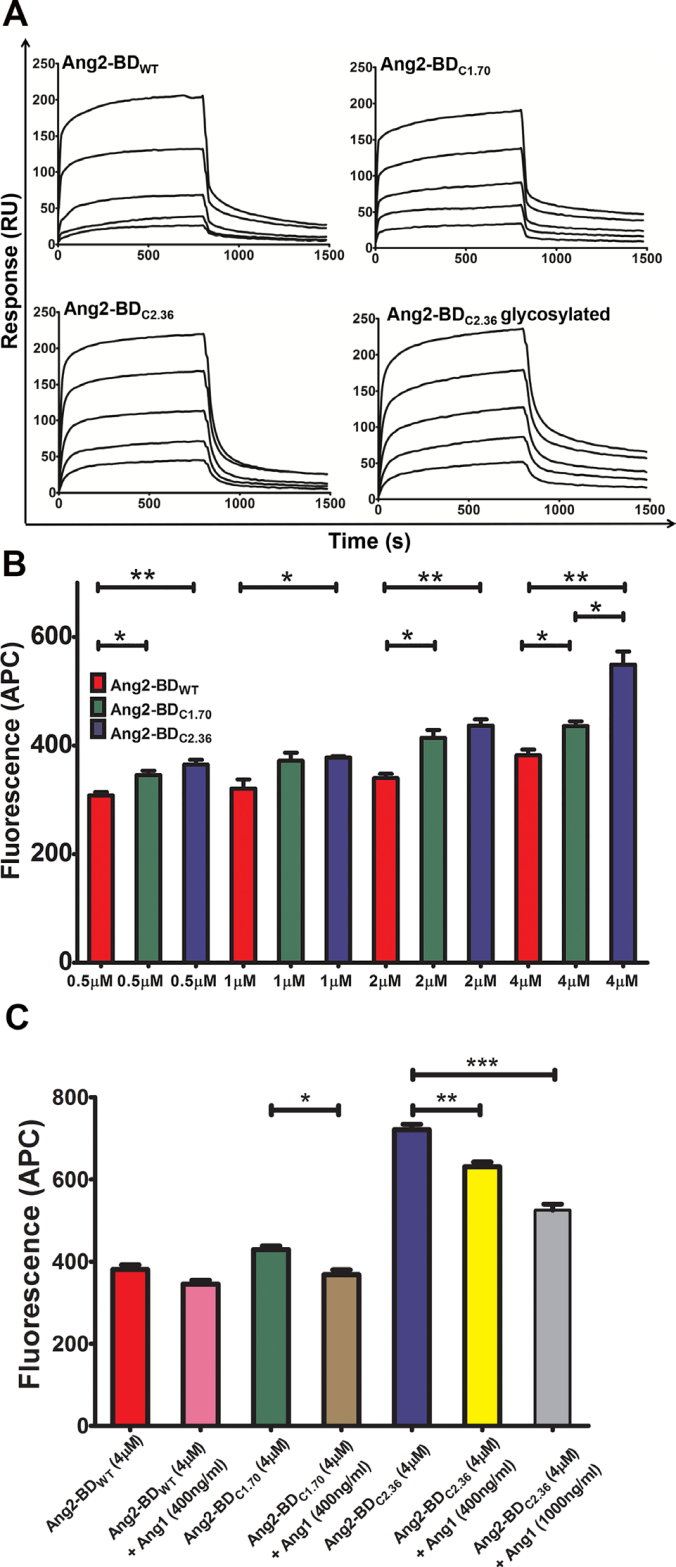
Binding of Ang2-BD variants to recombinant and cell-expressed human Tie2 (**A**) Representative SPR sensorgrams of binding of Ang2-BD variants to immobilized Tie2. The ranges of protein concentrations analyzed are indicated in parentheses: Ang2-BD_WT_ (18.75 nM – 300 nM); Ang2BD_C1.70_ (6.25 nM – 100 nM); Ang2-BD_C2.36_ (5 nM – 80 nM); Ang2-BD_C2.36_ glycosylated (6.25 nM – 100 nM). (**B**) Binding of Ang2-BD variants to TIME cell line: 1 ×10^5^ cells were incubated with indicated proteins (Ang2-BD_WT_, Ang2-BD_C1.70 and_ Ang2-BD_C2.36_, red, green and blue, respectively) for 2 h at 4°C with a gentle agitation. Mean fluorescence values were determined by flow cytometry using a fluorescently labeled antibody against a FLAG epitope tag. Data shown is the average of triplicate experiments, and error bars represent standard error of the mean. *indicates *P value* < 0.05 for comparison of results between Ang2-BD variants at the same concentration. (**C**) Competitive binding assay of Ang2-BD_WT_, Ang2-BD_C1.70_ and Ang2-BD_C2.36_, without Ang1 (red, green and blue, respectively) and with 400 ng/ml Ang1 (pink, brown and yellow, respectively) and 1000 ng/ml Ang1 for Ang2-BD_C2.36_ competition (grey). *indicates *P value* <0.05 for comparison of results between Ang2-BD variants with and without Ang1. Data shown is the average of triplicate experiments, and error bars represent standard error of the mean.

Ang2-BD_WT_ and the mutant variants were produced recombinantly in Pichia pastoris GS115 strain and purified using affinity chromatography, followed by treatment with endoglycosidase H (Endo Hf) to remove all N-linked carbohydrates (Supplementary Figure 4) and size-exclusion chromatography (SEC) (Supplementary Figure 5). Circular dichroism (CD) spectra revealed no change in structure of Ang2-BD_C1.70_ and Ang2-BD_C2.36_ variants in both their glycosylated and non-glycosylated forms in comparison to Ang2-BD_WT_ (Supplementary Figure 6A). Thermal denaturation of the purified proteins revealed that the melting temperature (Tm) of Ang2-BD_WT_ and Ang2-BD_C1.70_ was 49°C, and that of Ang2-BD_C2.36_ was 47°C (Supplementary Figure 6B). The binding kinetics of Ang2-BD variants to Tie2 were determined by surface plasmon resonance (SPR) (Figure 3A). The K_D_ values were found to be: Ang2-BD_WT_ 434±37 nM, Ang2-BD_C1.70_ 71.25±2.34 nM, and Ang2-BD_C2.36_ 42.61±0.77 nM and 51.45±1.36 nM for the non-glycosylated and the glycosylated forms, respectively (Table 1). The K_D_ values demonstrate an improvement in binding to Tie2 of 6-fold for the first-generation variant (Ang2-BD_C1.70_) and 10-fold for the second-generation variant (Ang2-BD_C2.36_). The kinetic parameters for the first- and second-generation variants are shown in Table 1. No significant difference in binding affinity was observed between the glycosylated and the non-glycosylated forms of Ang2-BD_C2.36_. The affinity of the purified Ang2-BD variants to Tie2 showed the same trend as that observed with the yeast displayed Ang2-BD variants, demonstrating the utility of YSD in quantitatively discriminating between clones that differ by as little as 6-fold (Ang2-BD_C1.70_ vs. Ang2-BD_WT_) in binding affinity to the desired target.

**Table 1:** Equilibrium binding affinities and kinetic rate constants for Ang2-BD variants to immobilized Tie2

Variant	SPR (immobilized Tie2)					
	Steady state	Two state binding model				
	*K_D_* ± SEM, nM	*K_D_* ± SEM, nM	*K_on1_* (M^−1^ s^−1^) × 10^5^	*K_off1_* (s^−1^) × 10^−2^	*K_on2_* (s^−1^) × 10^−3^	*K_off2_* (s^−1^) × 10^−4^
Ang2-BD_WT_	434 ± 37	-	-	-	-	-
Ang2-BD_C1.70_	*	71.25 ± 2.34	17.5 ± 0.01	12.1 ± 0.06	1.43 ± 0.01	5.62 ± 0.02
Ang2-BD_C2.36_	*	42.61 ± 0.77	7.71 ± 0.02	3.16 ± 0.01	1.23 ± 0.01	15.22 ± 0.06
Ang2-BD_C2.36_glycosylated	*	51.45 ± 1.36	5.59 ± 0.01	2.71 ± 0.01	1.73 ± 0.01	7.39 ± 0.03

-Values could not be accurately determined due to inadequate fit to two state binding model.

*Values could not be accurately determined from steady state model.

### Ang2-BD variants bind to cell-expressed Tie2

Binding of Ang2-BD variants to cell-expressed Tie2 was evaluated using the human telomerase-immortalized microvascular endothelium (TIME) cell line. These cells expressed Tie2 on their surface (Supplementary Figure 7), and Ang2-BD variants were found to bind to these cells in a dose-response manner (Figure 3B). Ang2-BD_C2.36_ exhibited the highest affinity towards TIME cells relative to the other Ang2-BD variant and to Ang2-BD_WT_. A competitive binding assay using Ang1 demonstrated that the Ang2-BD variants bind to Tie2 at the same epitope as does Ang1 (Figure 3C).

### Ang2-BD variants inhibit Tie2 phosphorylation of TIME cells

**Figure 4:**
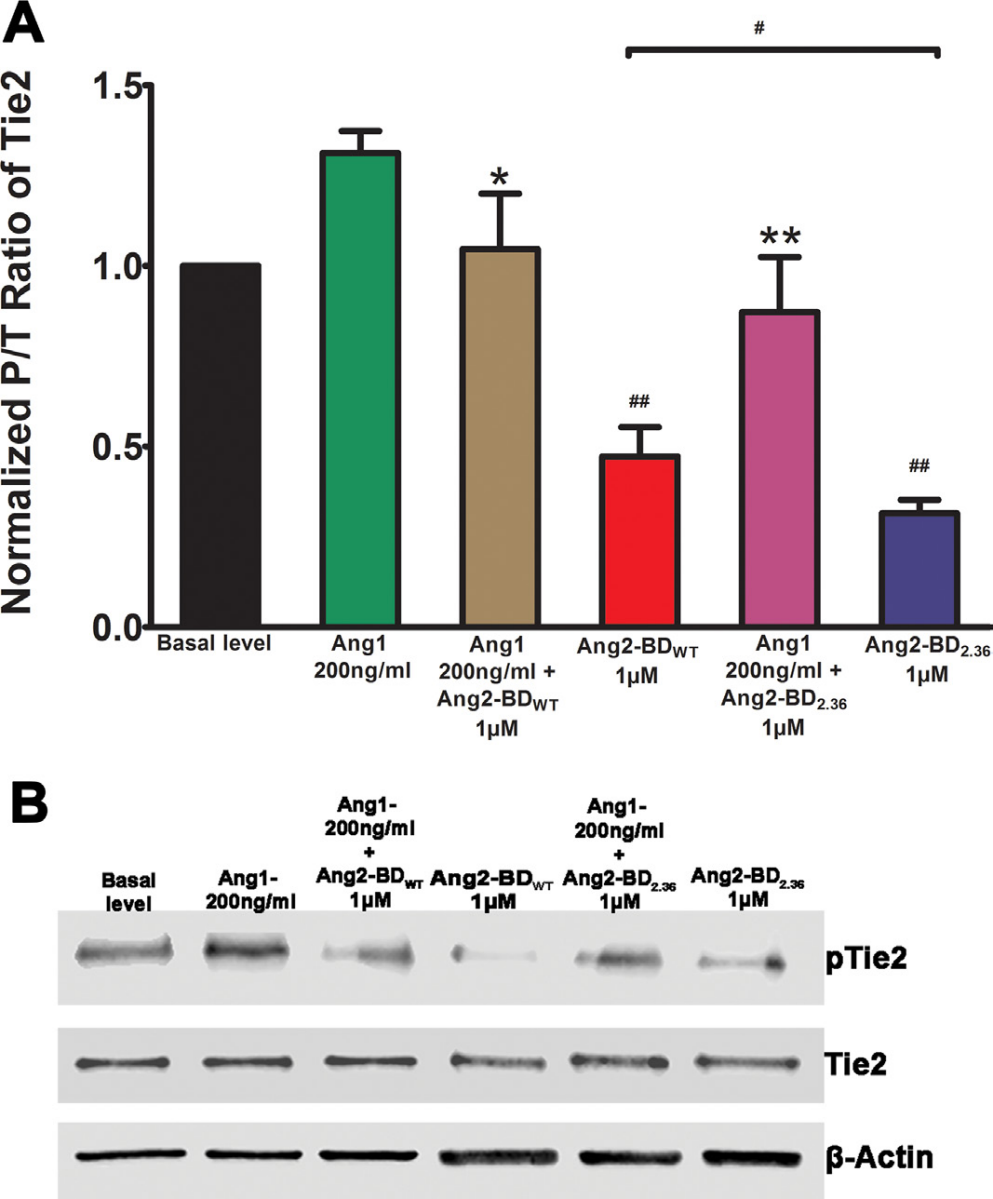
Inhibition of Tie2 phosphorylation by Ang2-BD variants (**A**) TIME cells were treated with control buffer (basal level, black), 200 ng/ml of Ang1 (green), 200 ng/ml of Ang1 and 1 μM Ang2-BD_WT_ (brown), 1 μM Ang2-BD_WT_(red), 200 ng/ml of Ang1 and 1 μM Ang2-BD_c2.36_ (blue) and 1 μM Ang2-BD_C2.36_ (purple) for 15 min for Tie2 phosphorylation. (**B**) Cell lysates were analyzed by western blot using antibodies against pTie2, Tie2 and β-actin. # indicates *P value* < 0.05 for comparison of results between Ang2-BD_WT_ and Ang2-BD_C2.36_; ## indicates *P value* < 0.01 for comparison of results between Ang1 + Ang2-BD_WT_ and Ang2-BD_WT_ and between Ang1 + Ang2-BD_C2.36_ and Ang2-BD_C2.36_; *indicates *P value* < 0.05 for comparison of results between Ang1 and Ang1 + Ang2-BD_WT_; **indicates *P value* < 0.01 for comparison of results between Ang1 and Ang1 + Ang2-BD_C2.36_. Data shown is the average of triplicate experiments, and error bars represent standard error of the mean.

To test the ability of the Ang2-BD variants to inhibit Tie2 phosphorylation, we used a system exploiting the endogenous expression of basal levels of Ang1, which induce phosphorylation of Tie2, in TIME cells. This phosphorylation is enhanced by the addition of soluble full-length Ang1 to the cell culture [8]. The results obtained demonstrate that the Ang2-BD variants could significantly inhibit Tie2 phosphorylation induced by both endogenous and soluble Ang1 and thus act as functional antagonists. In particular, the engineered high-affinity binder Ang2-BD_C2.36_ was found to be a more potent antagonist than Ang2-BD_WT_ (Figure 4).

### Ang2-BD variants inhibit tube formation and invasiveness of endothelial cells

**Figure 5:**
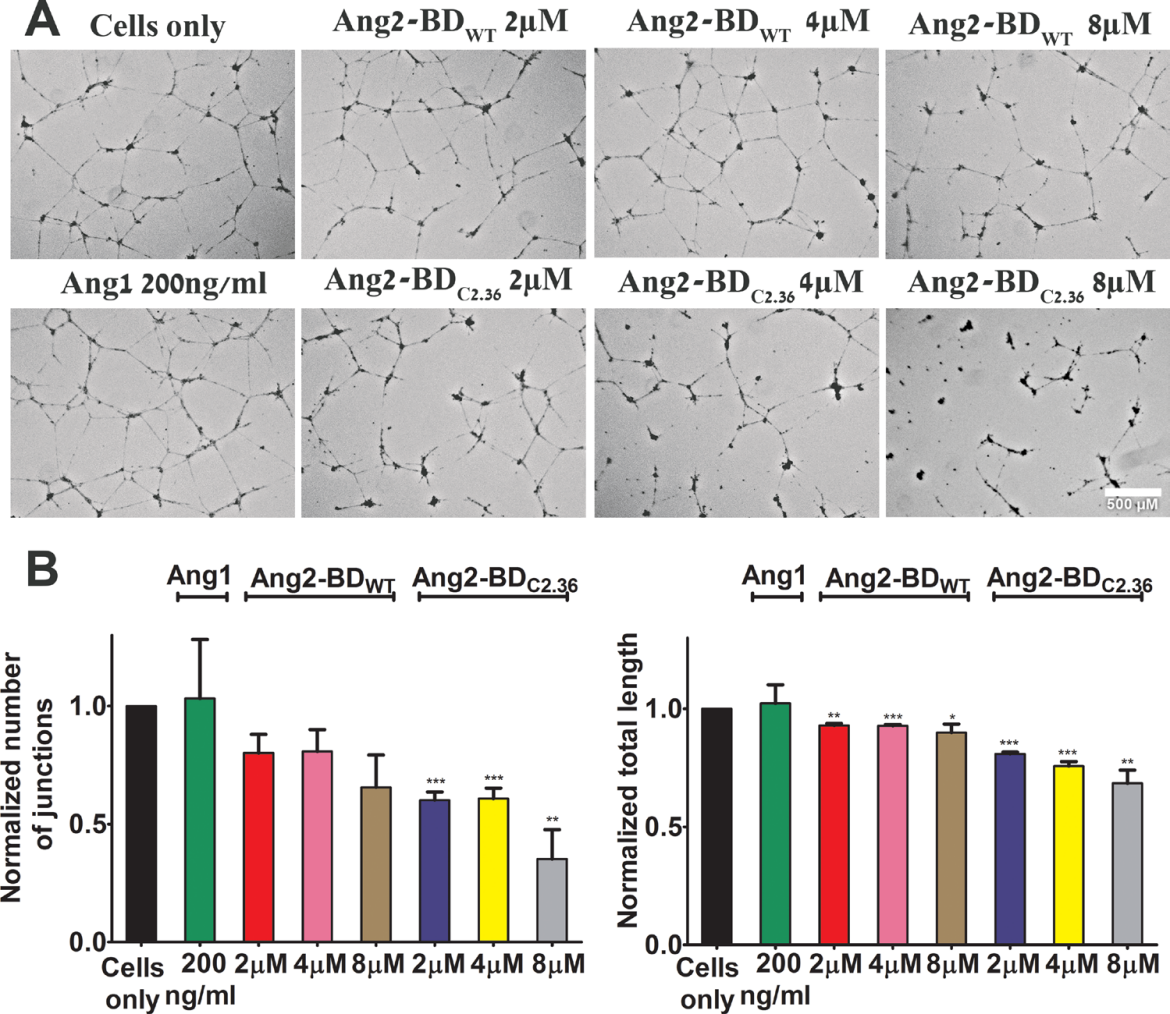
Inhibition of tube formation in endothelial cells by Ang2-BD variants (**A**) TIME cells were treated with the indicated proteins. (**B**) Control buffer (cells only, black), 200 ng/ml of Ang1 (green), 2 μM, 4 μM and 8 μM Ang2-BD_WT_ (red, pink and brown respectively), 2 μM, 4 μM and 8 μM Ang2-BD_C2.36_ (blue, yellow and grey respectively). Tube structures were analyzed for the number of generated junctions and the total tube length. *indicates *P value* < 0.05 for comparison of results between cells alone and tested proteins. Data shown is the average of triplicate experiments, and error bars represent standard error of the mean. Scale bar, 500 μm.

**Figure 6:**
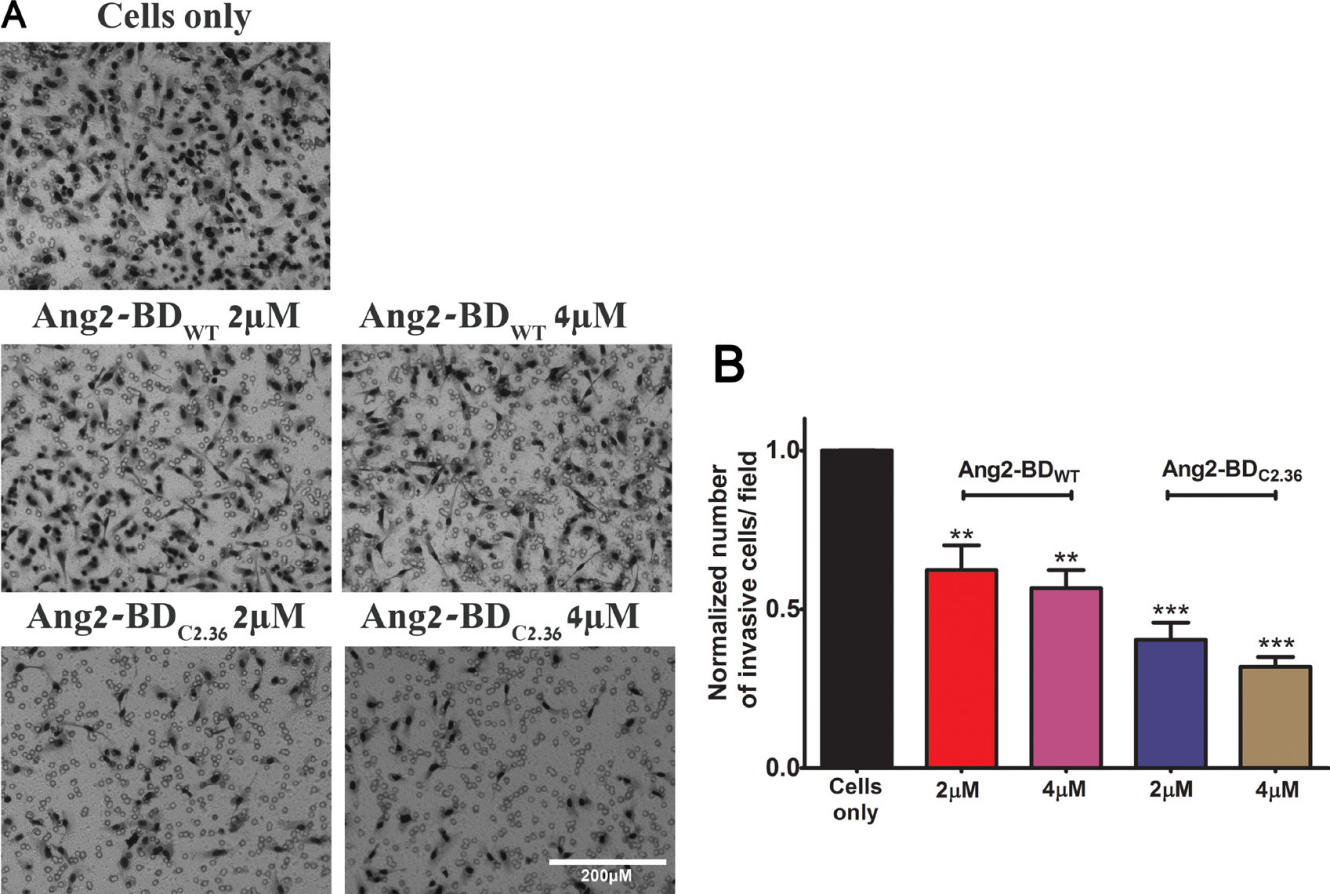
Inhibition of endothelial cells invasiveness by Ang2-BD variants (**A**) TIME cells were treated with indicated proteins in Boyden chambers. (**B**) Control buffer (cells only, black), 2 μM and 4 μM Ang2-BD_WT_ (red and pink, respectively), 2 μM and 4 μM Ang2-BD_C2.36_ (blue and brown, respectively). The invading cells accumulated on the bottom of membrane were counted in 16 frames for each membrane and analyzed for the number of cells. *indicates *P value* < 0.05 for comparison of results between cells only and cells + tested proteins. Data shown is the average of triplicate experiments, and error bars represent standard error of the mean. Scale bar, 200 μm.

Ang2-BD variants were tested for their ability to inhibit capillary tube formation by TIME cells grown on Matrigel, an extracellular basement membrane matrix. The Ang2-BD variants inhibited tube formation in a dose-dependent manner, and Ang2-BD_C2.36_ was found to be a potent inhibitor in comparison to Ang2-BD_WT_ (Figure 5). When Ang2-BD variants were tested for their ability to inhibit endothelial cells invasiveness, it was found that Ang2-BD_C2.36_ was superior to Ang2-BD_WT_ in inhibiting the invading cells (Figure 6).

## DISCUSSION

The success story of protein-based therapeutics centers on monoclonal antibodies [47, 48] that exert their activity either through immune-related effector functions or by inhibiting dysregulated ligand-receptor interactions. The above notwithstanding, the development of monoclonal antibodies that target Ang/Tie2 interactions has met with little success, and only a few human monoclonal antibodies that target the angiopoietin ligand (3.19.3 and MEDI3617) have entered early-stage clinical trials [28, 29]. The current study opens the way to a more promising approach, namely, a combinatorial methodology for engineering natural ligands to function as alternatives to antibodies. We have shown that this method is indeed an effective strategy for creating ligand-based RTK receptor inhibitors and molecular tools to study the effects of growth factor/RTK recognition on receptor activation and cell function.

Some success has indeed been achieved by applying rational methods relying on natural ligand-receptor interactions to the engineering of growth factor-based RTK antagonists [38, 44]. Nonetheless, despite the relative simplicity of these methods, the development of first-generation agents as therapeutics has met with several difficulties in terms of improving binding affinity, stability and expression. For example, a study designed to engineer vascular endothelial growth factor-A (VEGFA) from its natural dimeric form into a monomer showed that the binding affinity of the monomer for the ligand's VEGFR2 receptor was markedly less (by three orders of magnitude) than that of natural dimer [49]. Similarly, a number of studies have shown significantly decreased stability of single domain ligands engineered in isolation from their stabilizing domains [50, 51]. Finally, low levels of recombinant expression have been reported for multidomain mammalian proteins, such as growth factor ligands and RTKs with clinical potential [52, 53].

In a similar vein, a previous approach for transforming a multimeric Ang1 agonist into an antagonist, having two amino acid mutations corresponding to residues critical for Ang1 multimerization at the supercluster domain (SCD) of the molecules (i.e., C41S and C54S), produced an Ang1 variant that could not multimerize and was unable to activate Tie2, although it did bind well to Tie2 [3, 9, 45]. In addition, this recombinant monomeric Ang1 variant exhibited low expression yields and low stability [3, 45]. To overcome these limitations, particularly to generate new Ang-based variants with improved expression, stability and affinity to Tie2, we utilized combinatorial Ang2-BD libraries for generating protein diversity and YSD as a quantitative selection platform for enhancing expression, affinity and stability.

Sequencing of 70 and 60 clones selected from the final sorting rounds of the first and second generation Ang2-BD libraries, yielded 5 and 14 unique sequences for each library, respectively. None of the first generation Ang2-BD_C1.70_ mutations either reverted to wild-type residues or was replaced with other mutations during the second-generation library screens, demonstrating the strength and effectiveness of the YSD system in affinity maturation.

We found that two mutations, namely N467K and F469L, in the affinity-matured clones were common to the clones selected from the two independent libraries. This result supports the idea that these mutations, which are located in the Ang2-BD/Tie2 binding interface, are indeed involved in the improved affinity between Ang2-BD and Tie2, through direct binding interactions. The fact that three mutations were observed for position 467 (N467Y, N467H and N467K) further suggests that this position stabilizes the Ang2-BD/Tie2 complex. Another common mutation (already identified from the first-generation screens), namely, I413T, was located very close to the Ang2-BD/Tie2 binding interface [11] and could therefore also contribute to the conformational stability of the binding site (Figure 2).

The most commonly observed mutations fall into two classes: those located within the Ang2-BD/Tie2 binding interface (residues 421, 432, 434, 436, 467, 469, 470 and 475), and those located along the Ang2-BD backbone (residues 302, 304, 324, 330, 343, 353, 359, 386, 389, 393, 402, 407, 413 and 415). The most marked changes in amino acid properties were evident in mutations K432N, N467K, N467H, N470D, Y475H, and smaller changes, in N421Y, I434T and N467Y. The above mutations manifested as electrostatic changes (K432N, N467K, N467H, N470D and Y475H), as changes in the hydrophobicity (N421Y and N467Y), or as a combination of altered size and altered hydrophobicity (I434T).

Mutations K432N and I434T were clustered together in the first-generation clone Ang2-BD_C1.70_. It is possible that these mutations together contribute to binding. In the second-generation sorts, K436R, which lies in close proximity to K432N and I434T, was added to this cluster in clone Ang2-BD_C2.19_. Importantly, a comparison of the second-generation sequences allowed us to identify unique mutations that independently enhance the binding of Ang2-BD to Tie2. These include N421Y, N467K, N467Y and F469L; all had an individual effect on binding affinity.

Steady state and two-state binding models were used to obtain the best fit for the experimental SPR data for the respective Ang2-BD_WT_ and the engineered high affinity variants. The differences between the observed binding mechanisms may result from mutations within the calcium-binding loop (Figure 2), which may affect calcium binding and, as a result, the mode of interaction of Ang2-BD/Tie2 [11]. The binding of the engineered second-generation Ang2-BD mutant Ang2-BD_C2.36_ to Tie2 was approximately twice as strong as that of the first generation mutant Ang2-BD_C1.70_ and 10-fold stronger than that of the parental Ang2-BD_WT_. Glycosylation of Ang2-BD_C2.36_ did not have any influence on its affinity for Tie2, suggesting that the interaction between this variant and Tie2 was mediated solely by the amino acid residues of Ang2-BD_C2.36_. This finding is also in agreement with previous reported data for Ang2-BD_WT_ protein [11].

Ang2-BD_C2.36_ bound more strongly than Ang2-BD_WT_ to endothelial cells, with the binding being dose dependent. This variant was able to compete with full-length Ang1 for binding to endothelial cells, demonstrating direct interaction with cellular Tie2. Not surprisingly, the ability of Ang2-BD_WT_ and its Ang2-BD_C2.36_ variant to inhibit Tie2 phosphorylation in TIME cells was highly correlated with their affinity for recombinant soluble Tie2 and for cell-surface-expressed Tie2. As expected, both Ang2-BD_WT_ and its Ang2-BD_C2.36_ variant inhibited Tie2 phosphorylation, with effect of the variant being stronger. A similar inhibitory trend (but a weaker effect) was observed in the presence of exogenous agonistic Ang1, which served as a Tie2 inducer.

Having established that the affinity-matured Ang2-BD variants do indeed antagonize Tie2 in endothelial cells, we examined the effect of the Ang2-BD variants on the angiogenesis process in these cells. An *in vitro* endothelial tube formation assay of endothelial cells incubated with Ang2-BD variants on an extracellular basement membrane matrix showed Ang2-BD_C2.36_ to be a potent inhibitor of the formation of capillary-like structures. In a different assay, the Ang2-BD variants were tested for their ability to inhibit the invasiveness of endothelial cells. Here again, the ability of Ang2-BD_WT_ and the affinity-matured variant Ang2-BD_C2.36_ to inhibit the formation of capillary-like structures by endothelial cells and to inhibit endothelial cell invasiveness was correlated with the affinity of the variants to Tie2. The results of the phosphorylation and tube formation assays thus further define the roles of Ang1 and Ang2-BD as Tie2 agonists and antagonists, respectively.

In conclusion, our combinatorial engineering strategy has provided both new tools for studying the molecular mechanisms that mediate Ang- and Tie2-dependent angiogenesis and further insight into the sequence-structure-function triad of the antagonistic Ang mutants. Perhaps even more importantly, the promising findings support the use of our strategy as a template for engineering high-affinity agents from natural protein ligands to create functional protein antagonists against biomedical targets.

## MATERIALS AND METHODS

### Preparation of YSD Ang2-BD constructs and libraries

The construct for Ang2-BD_WT_ (amino acids 281 to 496) was obtained by custom gene synthesis (Integrated DNA Technologies). Amplification of the gene was performed using primers containing NheI and BamHI restriction sites and a 12-amino-acid linker (LPDKPLAFQDPS) to connect the C-terminus of the Ang2-BD_WT_ protein with a c-Myc epitope. The amplified gene was then introduced into the pCTCON yeast display vector (a generous gift from the laboratory of Dane Wittrup, MIT). The first-generation library was prepared using the Ang2-BD_WT_ construct as the template, and the library was generated by error-prone PCR and homologous recombination into *Saccharomyces cerevisiae* EBY100 cells, as previously described [54]. The library size was 6 × 10^6^ transformants, as estimated by dilution plating on selective SDCAA medium (2% dextrose, 1.47% sodium citrate, 0.429% citric acid monohydrate, 0.67% yeast nitrogen base and 0.5% casamino acids, pH 4.5). The second-generation library was prepared as described above using the Ang2-BD_C1.70_ clone, isolated from first-generation library sort 5, as the template. The library size was about 8 × 10^6^ transformants, as estimated by dilution plating on selective SDCAA medium.

### Screening of YSD Ang2-BD libraries

The yeast-displayed Ang2-BD libraries growing in selective SDCAA medium were induced for expression with 2% w/v galactose at 20°C overnight until an OD of 3.0 was reached, according to established protocols [54]. The first-generation library underwent five rounds of screening using high-throughput flow cytometric sorting to isolate clones with high affinity for recombinant human Tie2 (Ala23-Lys745). Initial and final sorts were performed using 100 nM and 5 nM Tie2-Fc, respectively. A diagonal sorting gate including 1% of the entire yeast pull was used to select Ang2-BD mutants that bind strongly to Tie2, relative their expression. For each round of sorting, yeast cells of approximately 10 times the library size were labeled as described below to facilitate fluorescent detection by flow cytometry. Binding of the yeast-displayed Ang-BD library to Tie2 was detected using soluble Tie2-Fc (R&D Systems) in Tie2 binding buffer [20 mM Hepes, pH 7.0, 150 mM NaCl, and 1% bovine serum albumin (BSA)], and Ang-BD expression levels were detected using 1:50 dilution of mouse anti c-Myc 9E10 antibody (Abcam) in a 1-h reaction at room temperature. Cells were washed and resuspended in ice-cold PBSA (phosphate buffered saline + 1% BSA) containing a 1:50 dilution of anti-human Fc fluorescein isothiocyanate (FITC) conjugated antibody (Sigma) and a 1:50 dilution of phycoerythrin (PE) conjugated anti-mouse IgG (Sigma). After 25 min on ice, yeast cells were washed in PBSA and sorted using iCyt Synergy FACS (fluorescence-activated cell sorting) [Proteomics Unit, National Institute for Biotechnology in the Negev (NIBN), Ben-Gurion University of the Negev (BGU)]. Plasmid DNA was extracted from the yeast clones using a Zymoprep kit (Zymo Research) and transformed into electrocompetent *Escherichia coli* cells for plasmid miniprep (RBC Bioscience Corp, Taiwan) and DNA sequencing (DNA Microarray and Sequencing Unit, NIBN, BGU). The second-generation library was subjected to five rounds of sorting using the method described above, where the initial and final sorts were performed with 20 nM and 500 pM Tie2, respectively. Sixty clones from the two final sorts were sequenced (DNA Microarray and Sequencing Unit, NIBN, BGU) and evaluated for their binding affinity towards Tie2-Fc by dividing the mean fluorescence intensity (MFI) of the Tie2 binding signal by the MFI of expression levels. The values obtained were normalized to Ang2-BD_WT_.

### Purification of Ang2-BD variants

The Multi-Copy *Pichia* Expression Kit (Invitrogen K1750–01) was used to produce the soluble proteins, as previously described [55]. The Ang2-BD variants were cloned into the pPIC9K vector for expression in *P. pastoris* yeast strain GS115 using EcoRI and AvrII restriction sites. Plasmid DNA (approximately 20 μg) was linearized by digestion with SacI (New England Biolabs) and electroporated into P. pastoris. Proteins were prepared with an N-terminal FLAG epitope tag and a C-terminal hexahistidine tag as handles for cell binding studies and protein purification, respectively. Transformed yeast cells were allowed to recover on RDB plates (18.6% sorbitol, 2% agar, 2% dextrose, 1.34% yeast nitrogen base, 0.001% biotin and 0.005% l-glutamic acid/l methionine/l-leucine/l-lysine/l-isoleucine) for two days at 30°C and were then selected for growth on YPD plates (1% yeast extract, 2% peptone and 2% dextrose) containing 4 mg∕mL Geneticin (Gibco). Several Geneticin-resistant colonies were grown in BMGY (1% yeast extract, 2% peptone, 0.23% potassium phosphate monobasic, 1.18% potassium phosphate dibasic, 1.34% yeast nitrogen base, 0.00004% biotin and 1% glycerol), followed by induction in BMMY (1% yeast extract, 2% peptone, 0.23% potassium phosphate monobasic, 1.18% potassium phosphate dibasic, 1.34% yeast nitrogen base, 0.00004% biotin and 1% methanol) for four days, with the methanol concentration being maintained at 1% throughout. Protein expression was detected by Western blot analysis of the culture supernatants, using an antibody against the FLAG epitope tag (Sigma). The highest expressing colony for each individual mutant was scaled up for expression by growing the yeast cultures in baffled base shake flasks. Ang2-BD variants were purified from yeast culture supernatants by metal chelating chromatography using a 5-ml HisTrap FF column (GE Healthcare) with 10 mM imidazole and eluted with 250 mM imidazole. Eluted protein fractions were concentrated, and the buffer was exchanged for 20 mM Hepes, pH 7.0, 150 mM NaCl buffer using a 10-kDa cutoff Vivaspin^®^ concentrator (GE Healthcare). Approximately 4 mg of purified protein were treated with Endo Hf (3,000 Units, New England Biolabs) overnight at room temperature to remove N-linked glycosylation. Gel filtration chromatography was performed using a Superdex 75 column (GE Healthcare) equilibrated with 20 mM Hepes, pH 7.0, 150 mM NaCl at a flow rate of 0.4 ml/min on an ÄKTA pure instrument (GE Healthcare). Proteins were analyzed by SDS-PAGE under non-reducing conditions. Protein concentrations were determined by UV-Vis absorbance at 280 nm and an estimated extinction coefficient of 66,500 M^−1^cm^−1^ for all Ang2-BD variants. The molecular weights of the purified proteins were determined using a MALDI-TOF REFLEX-IV (Bruker) mass spectrometer (Ilse Katz Institute for Nanoscale Science & Technology, BGU).

### Far-UV circular dichroism spectroscopy

CD spectra were recorded on a Jasco J-715 spectropolarimeter over a range of 185–260 nm in 20 mM Hepes, pH 7.0, 150 mM NaCl buffer using a quartz cuvette with a path length of 1 mm. Protein spectra were collected at a scanning speed of 50 nm/min and a data interval of 1 nm. Four scans of 30 μM protein solutions were averaged to obtain smooth data. All spectra were background corrected with respect to 20 mM Hepes, pH 7.0, 150 mM NaCl buffer and converted to units of mean residue ellipticity. For thermal denaturation studies, ellipticity was monitored at 230 nm using a 1°C/min scan rate.

### Surface plasmon resonance experiments

The binding interactions of Tie2 to Ang2-BD_WT_, Ang2-BD_C1.70_, Ang2-BD_C2.36_ (glycosylated and non-glycosylated) were analyzed (Proteomics Unit, NIBN, BGU) in real-time by SPR using a ProteOn XPR36 instrument (Bio-Rad). A ProteOn GLC sensor chip (Bio-Rad) was air initialized, and PBST (PBS×1, 0.005% Tween) buffer was flushed through the instrument prior to binding measurements. The rhTie2 extracellular domain (Sino Biological Inc.) was immobilized on the surface of a GLC sensor chip by using the amine coupling reagents N-hydroxysuccinimide (0.1 M; sulfo-NHS) and 1-ethyl-3-(3-dimethylaminopropyl)-carbodiimide (0.4 M; EDC; Bio-Rad). rhTie2 (0.8 μg) in 10 mM sodium acetate, pH 5.0, was allowed to flow over an activated GLC sensor chip channel surfaces, respectively, at a flow rate of 30 μL/min until the target immobilization level (2100 RU) was reached. BSA (3 μg) in 10 mM sodium acetate, pH 4.5, was then allowed to flow over the activated surfaces of a control GLC sensor chip channel at a flow rate of 30 μl/min until the target immobilization level (3000 RU) was reached. After protein immobilization, the chip surface was treated with 1 M ethanolamine HCl at pH 8.5 to deactivate excess reactive esters. All binding experiments were performed at 25°C in degassed Tie2 binding buffer (20 mM Hepes, pH 7.0, 150 mM NaCl, 1 mM CaCl_2_). A range of concentrations (18.7 nM to 300 nM for Ang2-BD_WT_, 6.25 nM to 100 nM for Ang2-BD_C1.70_, 5 nM to 80 nM for non-glycosylated Ang2-BD_C2.36_ and 6.25 nM to 100 nM for glycosylated Ang2-BD_C2.36_) of the protein analytes were allowed to flow over the surface-immobilized rhTie2 at a flow rate of 30 μl/min for 13.3 min, and the binding interactions were monitored. Following association, the dissociation of the various ligand-receptor complexes was monitored for 5 min. After the dissociation of each analyte, a regeneration step with 50 mM NaOH at a flow rate of 100 μl/min was performed. Each analyte sensorgram run was normalized by subtracting the BSA-immobilized channel and the zero analyte concentration runs. The binding constant (*K_D_*) was determined from the sensorgram of the equilibrium binding phase for Ang2-BD_WT_. Binding kinetics of Ang2-BD_C1.70_, glycosylated Ang2-BD_C2.36_ and non-glycosylated Ang2-BD_C2.36_ were analyzed by fitting to a two-state model.

### Cell binding assays

TIME cells (ATCC) were cultured in growth-factor-depleted Vascular Cell Basal Medium (ATCC) supplemented with 2% FBS and growth factor supplements (ATCC). For binding assays, 10^5^ cells were suspended in different concentrations of Ang2-BD variants in a total volume of 200 μl of PBSA (PBS and 0.1% BSA), followed by incubation at 4°C for 2 h with gentle agitation. Cell suspensions were centrifuged at 150 g at 4°C for 5 min and washed in 100 μl of PBSA followed by centrifugation at 150 *g* at 4°C for 5 min for two additional times. Cells were then resuspended in 100 μl of PBSA containing a 1:200 dilution of allophycocyanin (APC)-conjugated anti-FLAG antibody (Biolegend). After 20 min on ice, cells were washed twice in PBSA and analyzed by flow cytometry with a BD Accuri C6 flow cytometer (BD Biosciences). Mean fluorescence values were generated using FlowJo software (Treestar). For receptor level detection, 10^5^ cells were harvested, resuspended in 100 μl of PBSA with 1:100 APC-labeled anti-human Tie2 antibody (Biolegend), incubated at 4°C for 30 min, and then analyzed by flow cytometry. The data for the cellular assays was analyzed for column statistics with GraphPad Prism version 5.00 for Windows (La Jolla, CA, USA). Data shown is the average of triplicate experiments, and error bars represent standard error of the mean. Statistical significance was determined by column statistics and *t-test* analysis. *P value* < 0.05 was considered statistically significant.

### Tie2 phosphorylation assays

Confluent TIME cells were cultured in growth-factor-depleted Vascular Cell Basal Medium supplemented with 0.5% fetal bovine serum for 12 h at 37°C/5% CO_2_ prior to experimentation. Cells were then washed with PBS, and the medium was exchanged to fresh Vascular Cell Basal Medium depleted of growth factors and serum. After pretreatment with 1 mM sodium orthovanadate (Na_3_VO_4_, Sigma) for 15 min, cells were co-incubated for 15 min at 37°C with either commercial full-length rhAng1 as a positive control (R&D Systems) or a combination of full-length rhAng1 and the Ang2-BD variants. Since rhAng1 exists in different oligomeric states, the rhAng1 concentration was reported in mass concentration units instead of molar concentration units. Unstimulated cells were used as the negative control. Cells were then washed twice with PBS plus 1 mM Na_3_VO_4_ and lysed in ice-cold lysis buffer [20 mM HEPES, pH 7.4, 150 mM NaCl, 1% TritonX-100, 1 mM Na_3_VO_4_, and 1× complete protease inhibitor cocktail tablet (Roche)]. Cells were scraped from the culture plate wells, and the lysates were clarified by centrifugation (13,000 rpm for 30 min at 4°C). Protein concentration was measured by the BCA assay (Thermo Fisher Scientific), and equivalent amounts of each lysate sample were analyzed by duplicate 10% SDS-PAGE and transferred to duplicate PVDF membranes (Biorad). Blots were blocked (5% BSA, 50 mM Tris-HCl, pH 7.4, 150 mM NaCl, 0.1% Tween 20) for 1 h at room temperature and probed with a phospho-Tie2 specific rabbit polyclonal antibody (1:500 dilution; Y992–Tie2, R&D Systems) and a specific Tie2 rabbit monoclonal antibody (1:1000 dilution; Tie2 (D9D10) rabbit mAb, Cell Signaling Technology) overnight at 4°C. Membranes were washed three times with TBST (50 mM Tris-HCl, pH 7.4, 150 mM NaCl, 0.1% Tween 20) and probed with anti-rabbit, HRP-linked antibody (1:1000 dilution, Cell Signaling Technology) for 1 h at room temperature. Membranes were washed three times with TBST and then visualized and quantified using chemiluminescence (ECL, Biological Industries) and ImageJ software, respectively. The intensities of the phospho-Tie2 bands were adjusted for the expression of total Tie2 for each experiment. Blots were stripped and re-probed with anti-actin antibody for further normalization. Tie2 phosphorylation assay data was analyzed with GraphPad Prism version 5.00 for Windows (La Jolla, CA, USA). Data shown is the average of triplicate experiments, and error bars represent standard error of the mean. Statistical significance was determined by column statistics and t test analysis. *P value* < 0.05 was considered statistically significant.

### Endothelial cell tube formation assay

Serum-reduced Matrigel (10 mg/ml; BD Biosciences) was thawed overnight at 4°C, and 150 μl were added to each well of a 48-well microtiter plate and allowed to solidify for 1 h at 37°C. Wells were incubated with 3.25 × 10^4^ TIME cells with 2 μM, 4 μM and 8 μM of Ang2-BD_WT_ and Ang2-BD_C2.36_; rhAng1, 200 ng/ml, was added as the positive control. Cells were incubated for 16–18 h at 37°C/5% CO_2_. Cells were then washed twice in HBSS (Hanks’ balanced salt solution, Sigma), and capillary tube formation was observed using EVOS Cell Imaging Systems microscope (Thermo Fisher Scientific). Images were taken with EVOS 2× Objective, phase-contrast. Total length and number of junctions of the tubes were quantified by analysis of digitized images using ImageJ software and the Angiogenesis Analyzer plugin of the capillary-like structures. Tube formation assay data was analyzed with GraphPad Prism version 5.00 for Windows (La Jolla, CA, USA). Data shown is the average of triplicate experiments, and error bars represent standard error of the mean. Statistical significance was determined by column statistics and *t* test analysis. *P value* < 0.05 was considered statistically significant.

### Invasion assay

An *in-vitro* Boyden chamber assay was performed using ThinCert^™^ 24 well inserts (Greiner Bio-One). ThinCert cell culture insert membranes were coated with Matrigel (Corning) diluted in Vascular Cell Basal Medium (ATCC) to a 1:30 ratio. The lower compartment was filled with 600 μl of Vascular Cell Basal Medium supplemented with 2% FBS. TIME cells, 2 × 10^4^, with or without Ang2-BD variants, were incubated in 200 μl of supplement-free Vascular Cell Basal Medium, added to the pre-coated ThinCert cell culture inserts, and incubated for 20 h at 37°C with 5% CO_2_. Invasive cells were stained with DippKwik stain kit (American MasterTech Scientific) and were detected by EVOS FL Cell Imaging System at ×20 magnification. Quantification was accomplished by counting 16 fields for each membrane. Analysis of digitized images was performed using ImageJ software and Cell Colony Edge Analyser. Data was analyzed with GraphPad Prism version 5.00 for Windows (La Jolla, CA, USA). Data shown is the average of triplicate experiments, and error bars represent standard error of the mean. Statistical significance was determined by column statistics and t test analysis. *P value* < 0.05 was considered statistically significant.

## SUPPLEMENTARY MATERIALS FIGURES AND TABLES



## Abbreviations

PPI: protein–protein interaction
RTK: receptor tyrosine kinases
Ang: angiopoietin
Ang2-BD: angiopoetin 2 binding domain
YSD: yeast surface display
Endo Hf: endoglycosidase H
SEC: size-exclusion chromatography
CD: circular dichroism
SPR: surface plasmon resonance
TIME: telomerase-immortalized human microvascular endothelium
BSA: bovine serum albumin
FITC: fluorescein isothiocyanate
PE: phycoerythrin
FACS: fluorescence-activated cell sorting
NIBN: National Institute for Biotechnology in the Negev
BGU: Ben-Gurion University of the Negev
MFI: mean fluorescence intensity
APC: allophycocyanin
RU: response unit
