# Supplementary material for: Utilizing combinatorial engineering to develop Tie2 targeting antagonistic angiopoetin-2 ligands as candidates for anti-angiogenesis therapy
Source: Oncotarget. 2017 Apr 4;8(20):33571–85. doi: 10.18632/oncotarget.16827 (PMC5464891; doi:10.18632/oncotarget.16827)
Supplement: Supplementary file 1 [file oncotarget-08-33571-s001.pdf]

Utilizing combinatorial engineering to develop Tie2 targeting antagonistic angiopoetin-2 ligands as candidates for anti-angiogenesis therapy

Supplementary Materials

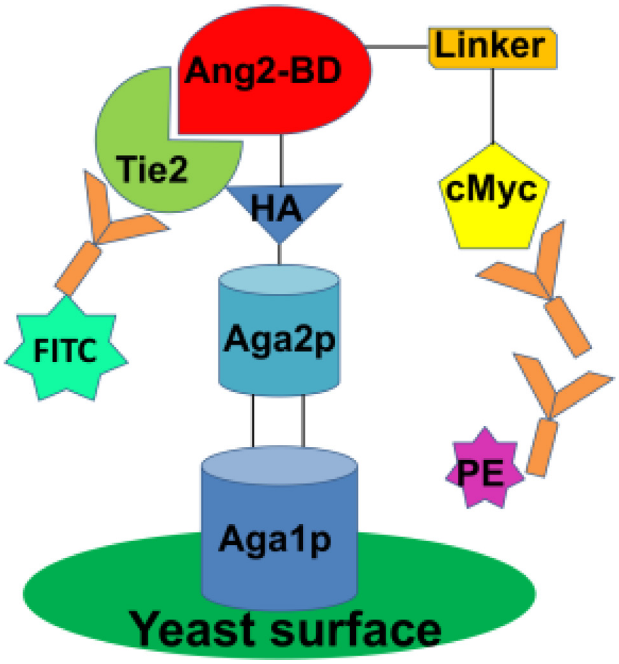

Supplementary Figure 1: The Ang2-BD construct displayed on yeast. Ang2-BD is presented on the cell surface of yeast as a fusion to agglutinin proteins. Display levels are detected using a primary antibody against the C-terminal cMyc tag (9E10 mouse antibody) and a phycoerythrin (PE)-labeled secondary anti mouse antibody. Binding to Tie2-Fc is measured using a fluorescein isothiocyanate (FITC) conjugated anti human Fc antibody.

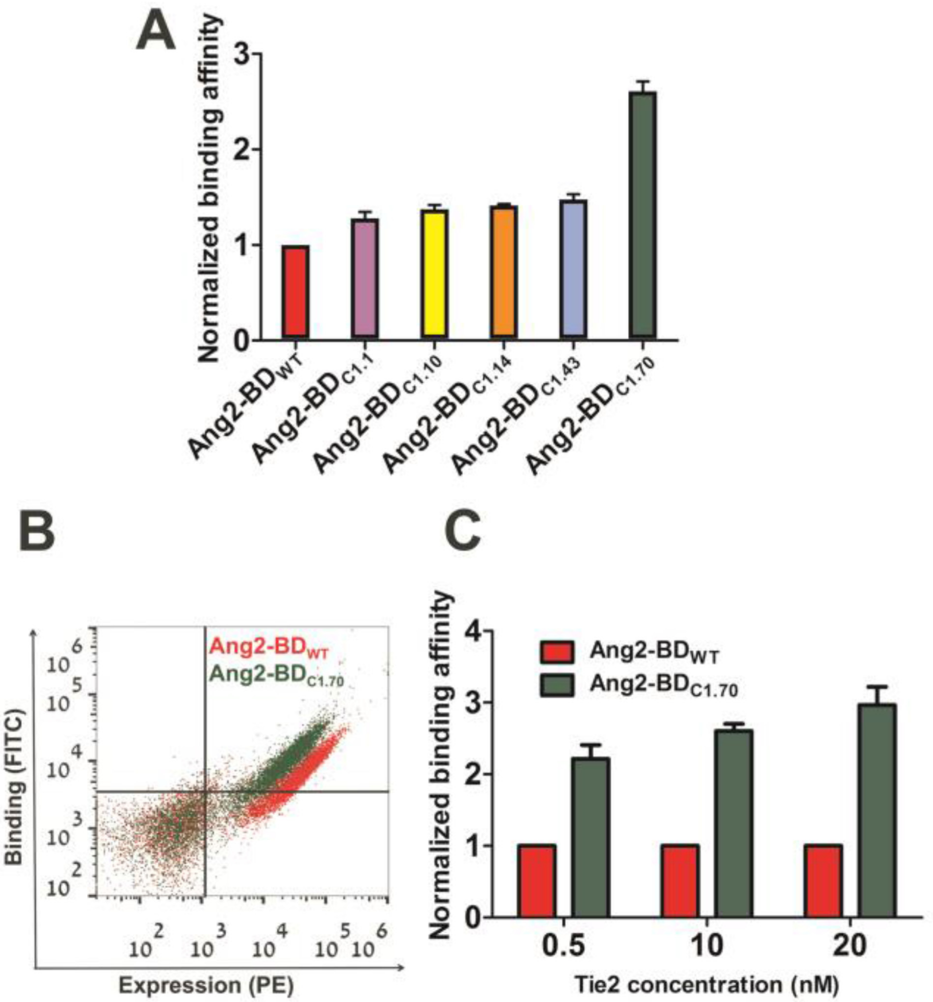

Supplementary Figure 2: Isolated clones with improved binding affinity towards Tie2. (**A**) Seventy individual clones were isolated from sort 5 of the Ang2-BD library, and their affinity (when displayed on yeast) for Tie2 (10 nM) was determined. Clones with improved affinity were sequenced with the aim to identify specific mutations. (**B**) FACS analysis of Ang2-BD_{WT} (red) and isolated clone Ang2-$BD_{C1.70}$ (green) binding to 10 nM Tie2. (**C**) Tie2 binding at different concentrations of Ang2-BD_{WT} (red) and isolated Ang2-$BD_{C1.70}$ (green). Data was normalized to the yeast surface expression levels of each clone and to Ang2-BD_{WT} binding affinity.

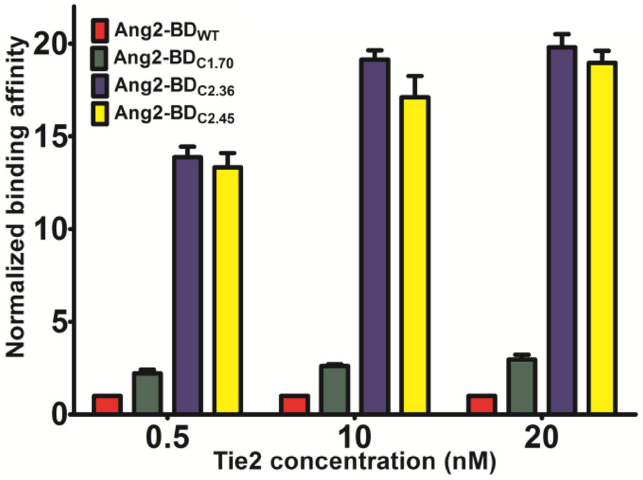

Supplementary Figure 3: Clones isolated from the second-generation library, with improved binding affinity towards Tie2. Sixty individual clones were isolated from sort 5 of the Ang2-BD$_{C1.70}$-based second-generation library, and their affinity for Tie2 at different concentrations was determined. Data was normalized to the yeast surface expression levels of each clone and to Ang2-BD$_{WT}$ binding affinity.

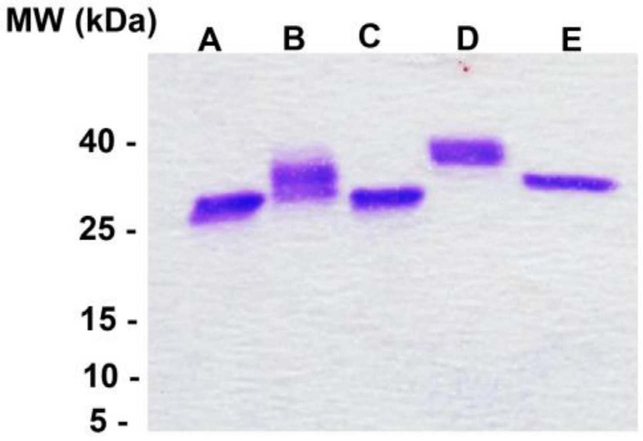

Supplementary Figure 4: Ang2-BD purification process. SDS PAGE gel showing the purified Ang2-BD$_{WT}$ (lane A), Ang2-BD$_{C1.70}$ untreated (lane B) and treated with Endo Hf (lane C), Ang2-BD$_{C2.36}$ untreated (lane D) and treated with Endo Hf (lane E). The molecular weights are 27.18 kDa for C1.70 and 27.2 kDa for C2.36.

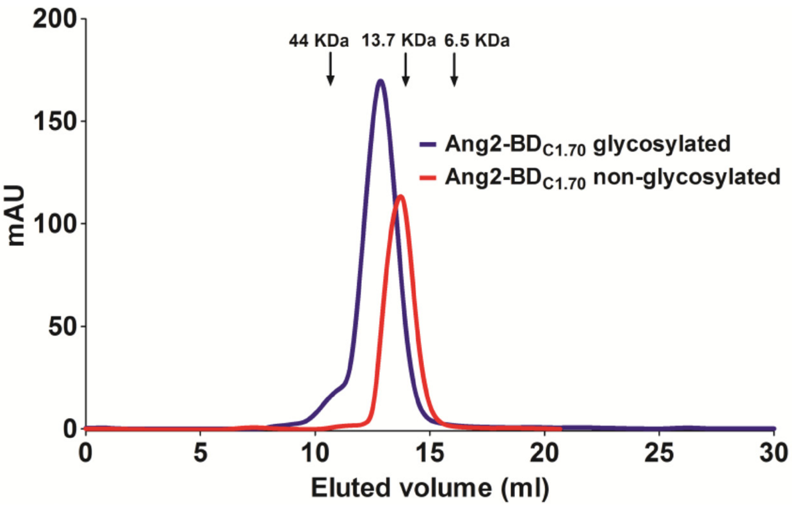

Supplementary Figure 5: Size exclusion chromatography (SEC) purification of Ang2-BD$_{C1.70}$. Glycosylated form is shown in blue, and non-glycosylated form in red. Elution volumes for the standards ovalbumin (44 kDa), ribonuclease A (13.7 kDa) and aprotinin (6.5 kDa) are shown. MS analysis of the molecular weights of the purified proteins gave: Ang2-BD$_{C1.70}$ glycosylated 32.7 kDa; Ang2-BD$_{C1.70}$ nonglycosylated 28.1 kDa; Ang2-BD$_{C2.36}$ glycosylated 33.2 kDa; Ang2-BD$_{C2.36}$ nonglycosylated 28.3 kDa; and Ang2-BD$_{WT}$ 27.7 kDa (Ang2-BD$_{WT}$ wild type was not glycosylated).

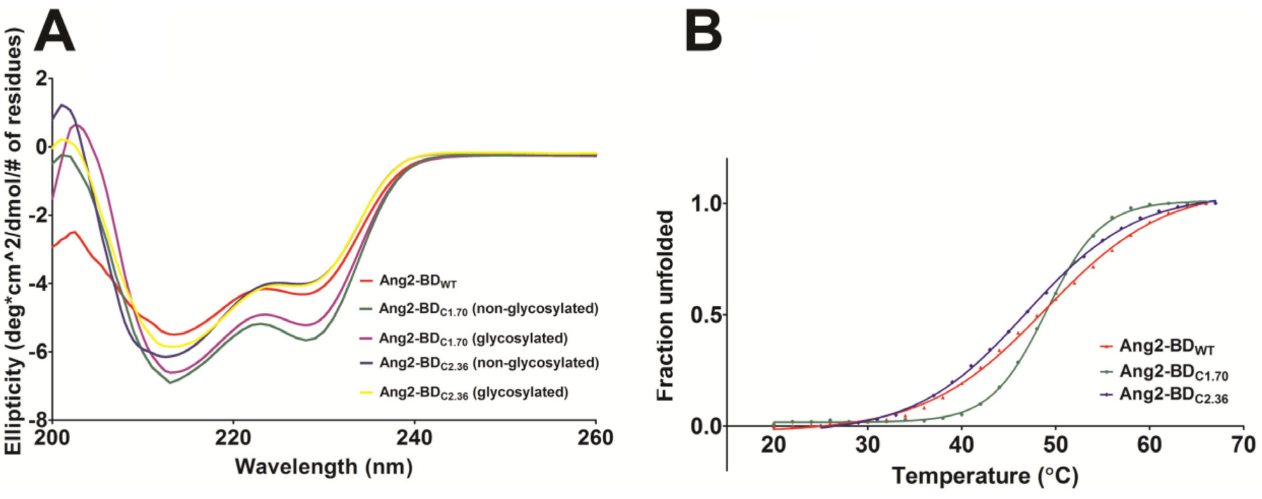

Supplementary Figure 6: Structural characterization and thermal denaturation of Ang2-BD variants. (**A**) CD spectra of Ang2-BD$_{WT}$ and Ang2-BD$_{C1.70}$ and Ang2-BD$_{C2.36}$ in both their glycosylated and non-glycosylated forms. (**B**) Thermal denaturation of purified Ang2-BD and the two Ang2-BD variants.

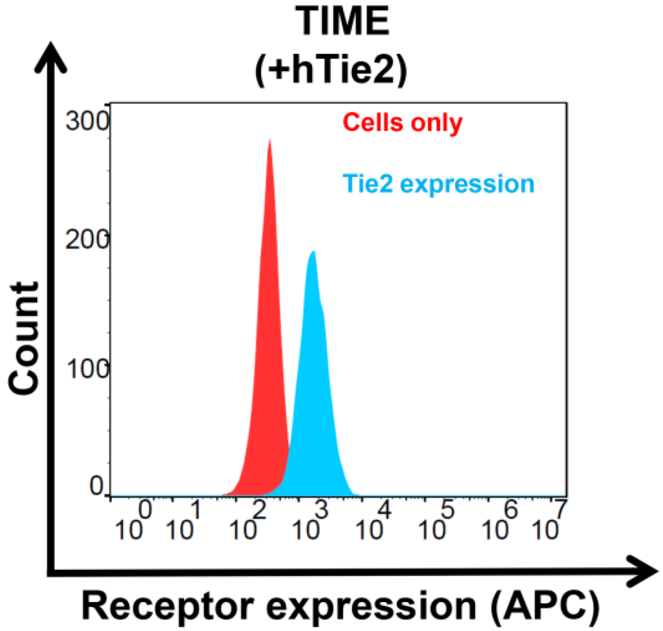

Supplementary Figure 7: Expression of Tie2 receptor on TIME cells. Levels of cell surface expression of Tie2 receptor (stained with APC-labeled anti-human Tie2, blue histogram) versus cells only control (unstained, red histogram), on TIME cells. 1×10^5 cells were either stained with APC-labeled anti-human Tie2 or unstained and incubated at 4 °C for 30 min and analyzed by flow cytometry.

Supplementary Table 1: Sequencing analysis of the isolated clones with improved binding affinity towards Tie2

	Residue number									Binding Interface Residues					Normalized binding for Tie2 (10 nM) (relative to Ang2-BDWT)
Variant	304	330	343	353	386	389	407	413	432	434	467	469	470	475	
Ang2-BDWT	N	D	K	Y	L	H	T	I	K	I	N	F	N	Y	**1**
C1.1					S							L		H	**1.3**
C1.10								T			K				**1.4**
C1.14				F			A			T					**1.4**
C1.43	D	N	E			R									**1.5**
C1.70								T	N	T			D		**2.5**
Number of repetitions	1	1	1	1	1	1	1	2	1	2	1	1	1	1	

Individual clones isolated after sort 5 were sequenced, and mutations at the Ang2-BD/Tie2 interface were identified (red).

Supplementary Table 2: Sequencing analysis of the isolated clones from the second generation library with improved binding affinity towards Tie2

	Residue number											
**Variant**	**302**	**324**	**359**	**393**	**402**	**421**	**436**	**467**			**469**	**Normalized binding for Tie2 (10 nM) (relative to Ang2-BDWT)**
**Ang2-BDWT**	F	I	F	S	H	N	K		N		F	1
**Ang2-BD$_{C1.70}$**	F	I	F	S	H	N	K		N		F	2.5
**C2.1**						Y						10.8
**C2.5**								Y				10.1
**C2.9**									H			8.38
**C2.10**	L							Y				10.5
**C2.12**					R	Y						12
**C2.16**			L					Y				11.7
**C2.19**							R			K		11.6
**C2.23**		T				Y						9.7
**C2.28**				L						K		6.68
**C2.36**						**Y**					**L**	17.1
**C2.37**	S					Y						12
**C2.45**										**K**		16.5
**C2.52**		T									L	9.23
**C2.59**											L	7.47
**Number of repetitions**	**1 + 1**	**2**	**1**	**1**	**1**	**5**	**1**	**3**	**1**	**3**	**3**	

Individual clones from the Ang2-BD$_{C1.70}$ based library were sequenced and their binding affinity towards Tie2 was tested.
